# Dr. Alexander Monro

**Published:** 1859-05

**Authors:** 


DR. ALEXANDER MONRO, late Emeritus Professor of Anatomy in the University of Edinburgh.—We regret to have to record the death of this eminent physician, which took place on the 10th inst., at his seat, Craiglockhart House, near Edinburgh. The deceased was the third of his family who, in direct succession, so ably filled the Anatomical Chair in the University of Edinburgh for upwards of a century. He was born November 5th, 1773; was educated at the High School; and studied at the University under the late Professors James Gregory, Cullen, Black, Duncan, etc. After having passed through a full course of medical education, and obtained the degree of M.D., he went to London to prosecute the study of anatomy and surgery under the famous Wilson, and from thence he repaired for a short time to Paris. In 1800 he was appointed conjoint Professor with his father, at whose death, in 1817, he became sole Professor of Anatomy and Surgery, and continued to lecture till 1846, when he resigned the chair. During the long period he delivered lectures his class was well attended. Among his pupils there are not a few who have risen to the highest eminence in medical science. Such names as Professors Alison, Traill, Christison, Syme, Liston, Miller, Edward Forbes, etc., and Drs. Abercrombie, Hope, Holland, Bright, Marshall Hall, and Sir Humphrey Davy, are of world-wide reputation.

Dr. Monro was the author of several anatomical, medical, and surgical treatises, of which the following are the principal: "Elements of Anatomy;" "Morbid Anatomy of the Brain;" "Morbid Anatomy of the Stomach, Gullet, and Intestines;" "Anatomy of the Perinæum;" "Anatomy of Crural Hernia;" etc. etc.

Dr. Monro was twice married: first, in 1800, to the eldest daughter of Dr. Carmichael Smyth, by whom he had six sons (one of whom died in infancy) and six daughters; secondly, in 1836, to a daughter of David Hunter, Esq., of London, who survives him.—*London Lancet*, March 26, 1859.

